# A Cross-Sectional Survey on Generation Z Physicians: Professional Value, Educational Expectations, and Work Environment Preferences

**DOI:** 10.7759/cureus.83317

**Published:** 2025-05-01

**Authors:** Kasumi Satoh, Yuki Mitani, Masahiro Ishikane, Shuhei Kamada

**Affiliations:** 1 Emergency and Critical Care Medicine Department, Akita University Graduate School of Medicine, Akita, JPN; 2 Emergency and Critical Care Medicine Department, Graduate School of Biomedical and Health Sciences, Hiroshima University, Hiroshima, JPN; 3 Disease Control and Prevention Center, National Center for Global Health and Medicine, Japan Institute for Health Security, Tokyo, JPN; 4 Urology Department, Chiba University Graduate School of Medicine, Chiba, JPN

**Keywords:** graduate medical education (gme), health professional's education, professionalism, value of life, workplace

## Abstract

Generation Z (Gen Z) physicians, born between the mid-1990s and the mid-2010s, bring unique professional values and expectations to clinical practice shaped by technological and socioeconomic changes. This study explored these aspects of Gen Z physicians in Japan. A cross-sectional survey focusing on professional values, job-related training, supervisor attitudes, and work environments was conducted among first-year to third-year physicians in Japan. The participants were categorized into the Gen Z (≤29 years old) and non-Gen Z groups. Wilcoxon rank-sum tests identified significant differences between the groups. Of the 429 respondents, 391 were Gen Z members. They value job satisfaction, work for the benefit of others, embrace new challenges, and desire immediate feedback. Notable differences between Gen Z and non-Gen Z members were observed based on median Likert scale scores (1 = strongly agree and 5 = strongly disagree). Specifically, Gen Z physicians more strongly agreed that work is primarily a means to earn money for their personal life (median of Gen Z = 2 {interquartile range {IQR}: 2-3} versus non-Gen Z = 2.5 {IQR: 2-4}; p < 0.01), preferred working as a team rather than individually (median of Gen Z = 2 {IQR: 2-3} versus non-Gen Z = 3 {IQR: 2-4}; p = 0.02), and desired immediate feedback (median of Gen Z = 1 {IQR: 1-2} versus non-Gen Z = 2 {IQR: 1-2}; p < 0.01). They also showed a stronger preference for proactive communication from supervisors (median of Gen Z = 2 {IQR: 1-2} versus non-Gen Z = 2 {IQR: 2-3}; p < 0.01), chat-based workplace communication (median of Gen Z = 2 {IQR: 1-3} versus non-Gen Z = 3 {IQR: 2-4}; p = 0.01), cooperation over competition with peers (median of Gen Z = 2 {IQR: 1-2} versus non-Gen Z = 2 {IQR: 2-3}; p = 0.01), and socializing with colleagues of the same age (median of Gen Z = 2 {IQR: 1-3} versus non-Gen Z = 2 {IQR: 2-3}; p = 0.04).

Japanese Gen Z physicians prioritize internal motivation, economic stability, immediate feedback, digital technological integration, and collaborative work environments. These findings necessitate adapted approaches to medical education and workplace settings that align with the distinct outlook of Gen Z.

## Introduction

Generational differences regarding values, behaviors, and identity often lead to conflicts among age cohorts in the workplace [1]. These differences may exist in the general workforce and the medical education and training of junior physicians. Value conflicts can arise between educators and learners of different generations, particularly during the training of young physicians. Generation Z, often called “Gen Z,” represents individuals born between the mid-1990s and the mid-2010s [2-5]. This generation is entering clinical practice as physicians, and it is necessary to discuss how to engage with them.

Previous research suggests that Gen Z high school students who aspire to attend medical school emphasize intrinsic motivation, altruism, and achievement [2]. It was suggested that the values emphasized by Gen Z in general, not just healthcare professionals, include helping others and idealism rather than self-centered interests [6,7]; these characteristics closely align with core aspects of professionalism. In addition, Gen Z has a unique mindset, having grown up in an era of digitalization and socioeconomic instability [4]; therefore, it is necessary to discuss learning environments and interpersonal relationships in professional education that suit them.

As they are still new to clinical practice, the values and work environments that Gen Z physicians value are unclear; however, understanding the professional characteristics of Gen Z is necessary to interact with them effectively. This study aims to determine the professional values and work environment preferences of Gen Z physicians.

This article was previously posted to the Research Square preprint server on December 28, 2023 (DOI: 10.21203/rs.3.rs-3766309/v1).

## Materials and methods

Ethics

Although our study falls outside the scope of the Ethical Guidelines for Medical and Health Research Involving Human Subjects (Ministry of Health, Labour and Welfare, Japan), the ethics committee of the Akita Medical Association approved this study (clinical research protocol number: 51). Informed consent was obtained from all participants after explaining at the beginning of the survey that their anonymized results would be used for academic purposes.

Study design and participants

This cross-sectional survey was conducted among physicians in their first to third clinical years using an electronic form based on the Checklist for Reporting Results of Internet E-Surveys (CHERRIES) [8]. The definition of Gen Z varies by source, and we define Gen Z in this study as those who were 29 years old or younger as of April 2023, that is, those born in 1994 or later [2-5]. The sample frame was a convenience sample of physicians registered with Medical Principles, Inc., in Tokyo. This survey was part of the data collection conducted by the corporation. The closed survey was introduced to physicians, and the participants were recruited via a member-only networking service. The sample size was set to approximately 400 because of budget constraints. The incentive for participation was a gift card worth 500 Japanese yen per person. The survey commenced on April 25, 2023, and was concluded as soon as the target number of responses was reached, which took two days.

Survey development and pretesting

The questionnaire consisted of four domains. The first domain asked for information about preferred values (eight items), the second domain asked about expected job-related education (six items), the third domain asked about attitudes expected of supervisors (four items), and the last domain asked about the preferred work environment (six items). To assess the views toward these factors, we used a five-point Likert scale (ranging from 1 = strongly agree to 5 = strongly disagree). The detailed questionnaire contents in English are presented in Table 1. The survey was conducted via Questant, provided by Macromill, Inc. (Tokyo, Japan). In the pilot phase, feedback was sought from nine young volunteers. They reviewed the clarity of each question to evaluate the usability and comprehensibility of the content.

**Table 1:** List of questionnaire presented to the participants.

Questionnaire		
Domain 1. Professional value emphasis	Q1	I value job satisfaction in my work.
	Q2	I value growth more than leisure in my job.
	Q3	I want to work for the benefit of others.
	Q4	Work is a means to earn money for my personal life.
	Q5	I want to face new challenges.
	Q6	I prefer to work as a team rather than individually.
	Q7	I want to achieve results, even if it means outperforming others.
	Q8	In the future, I would like to do something other than being a doctor.
Domain 2. Anticipated job-related training	Q1	I would actively participate in simulation training opportunities.
	Q2	I would like immediate feedback.
	Q3	I would like to be praised in front of many people.
	Q4	I only want to learn what I think is necessary for my future.
	Q5	I prefer to be taught hands-on rather than learning from practice.
	Q6	I do not mind working overtime if I feel it will help me grow.
Domain 3. Supervisor attitude expectations	Q1	I would like to be able to use emoticons and exclamation marks freely in professional communication with senior doctors.
	Q2	I enjoy company parties, including those with senior doctors.
	Q3	I value the ability to respect senior doctors more than their accessibility.
	Q4	I want the supervisor doctors to be proactive in communication.
Domain 4. Preferred work environment	Q1	I want to share personal stories with people in the hospital.
	Q2	I often experience a lack of digitalization at work.
	Q3	I prefer to communicate via “chat” rather than email for work-related contacts.
	Q4	With physicians my age, I value cooperation over growth through competition.
	Q5	I want to work where a patient-first attitude is always required.
	Q6	I enjoy socializing with colleagues and people my age.

Statistical analysis

All items were mandatory; only completed questionnaires were analyzed, and only data with complete responses to all survey items were submitted. The participants were divided into two groups for analysis: those aged up to 29 were classified into the Gen Z group. In contrast, those aged 30 and above were classified into the non-Gen Z group. Categorical variables describing participant characteristics (e.g., gender, age category, and training grade) were compared between the Gen Z and non‑Gen Z groups using Pearson’s chi‑square test; Fisher’s exact test was applied when any expected cell count was <5. The tables report the test statistic (χ²) and its corresponding two‑sided p‑value for these demographic comparisons. Survey results were presented using a Likert scale and compared between the two age groups (Gen Z versus non-Gen Z) as continuous variables using the Wilcoxon rank-sum test. Furthermore, the survey results were examined for gender differences within the Gen Z group using the Wilcoxon rank-sum test. The normality of continuous variables was tested using the Shapiro-Wilk test. All continuous variables demonstrated a skewed distribution and were summarized as median and interquartile range (IQR). All statistical analyses were performed using Stata® software version 18.0 (StataCorp LLC, College Station, TX) and R software version 4.3.3 (R Foundation for Statistical Computing, Vienna, Austria). Statistical significance was defined as a two-sided p-value of <0.05.

## Results

**Figure 1:**
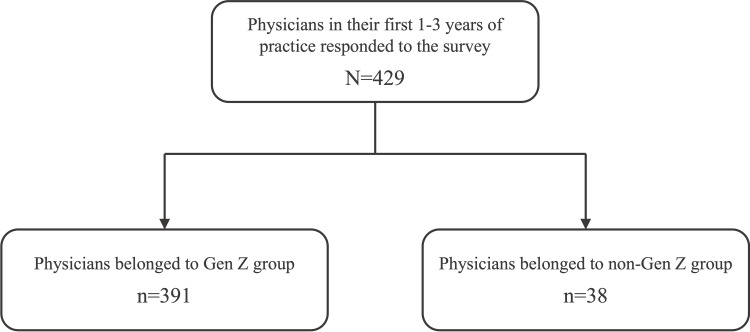
Flowchart representing the study participant selection process. The initial pool consisted of 429 physicians within their first 1-3 years of practice who responded to the survey. Out of this group, 391 physicians were identified as belonging to the Generation Z (Gen Z) group and 38 physicians belonging to the non-Generation Z (non-Gen Z) group.

A total of 429 physicians in their first to third year of practice responded to the questionnaire. Of these, 391 were classified into the Gen Z group and 38 into the non-Gen Z group (Figure 1). In the Gen Z group, approximately 60% were male participants, similar to the gender ratio of recent national medical examination passers. Approximately 70% of the participants were between 24 and 29 years old, and the majority were interns one to two years after post-graduation. The percentage of male participants in the non-Gen Z group was 10% higher than in the Gen Z group (Table 2).

**Table 2:** Demographic information of the participants per group (N = 429). This table presents the demographic information of the study participants who were divided into two groups: the Generation Z (Gen Z) group and the non-Generation Z (non-Gen Z) group. The demographic variables include age, gender, and grade. Values are n (%). Group differences were analyzed with Pearson’s chi‑square test for gender and PGY grade and with Fisher’s exact test for age categories. The “test statistic” column shows χ² values; for Fisher’s exact test, only the p‑value is reported. PGY: post-graduate year

Variables	Overall (N = 429)	Gen Z (N = 391)	Non-Gen Z (N = 38)	Test statistic	P‑value
Gender (n, %)				χ² = 1.51	0.22
Male	277 (64.57)	249 (63.68)	28 (73.68)		
Female	152 (35.43)	142 (36.32)	10 (26.32)		
Age (years; n, %)				-	<0.01
20-24	85 (19.81)	85 (21.74)	0 (0)		
25-29	306 (71.33)	306 (78.26)	0 (0)		
30-34	19 (4.43)	0 (0)	19 (50.00)		
35-39	14 (3.26)	0 (0)	14 (36.84)		
40-44	5 (1.17)	0 (0)	5 (13.16)		
Grade (n, %)				χ² = 8.58	<0.01
Intern (PGY-1,2)	328 (76.46)	249 (63.68)	15 (39.47)		
Resident (PGY-3)	101 (23.54)	142 (36.32)	23 (60.53)		

Questions that received a high proportion of “strongly agree” or “agree” responses included the following: In Domain 1, more than 80% of the participants agreed with Q1 and Q5, and more than 70% agreed with Q3. Similarly, in Domain 2, more than 80% of the participants agreed with Q1 and Q2; in Domain 3, more than 80% agreed with Q4. Finally, in Domain 4’s Q2, Q4, and Q6, more than 70% of the participants agreed.

**Figure 2:**
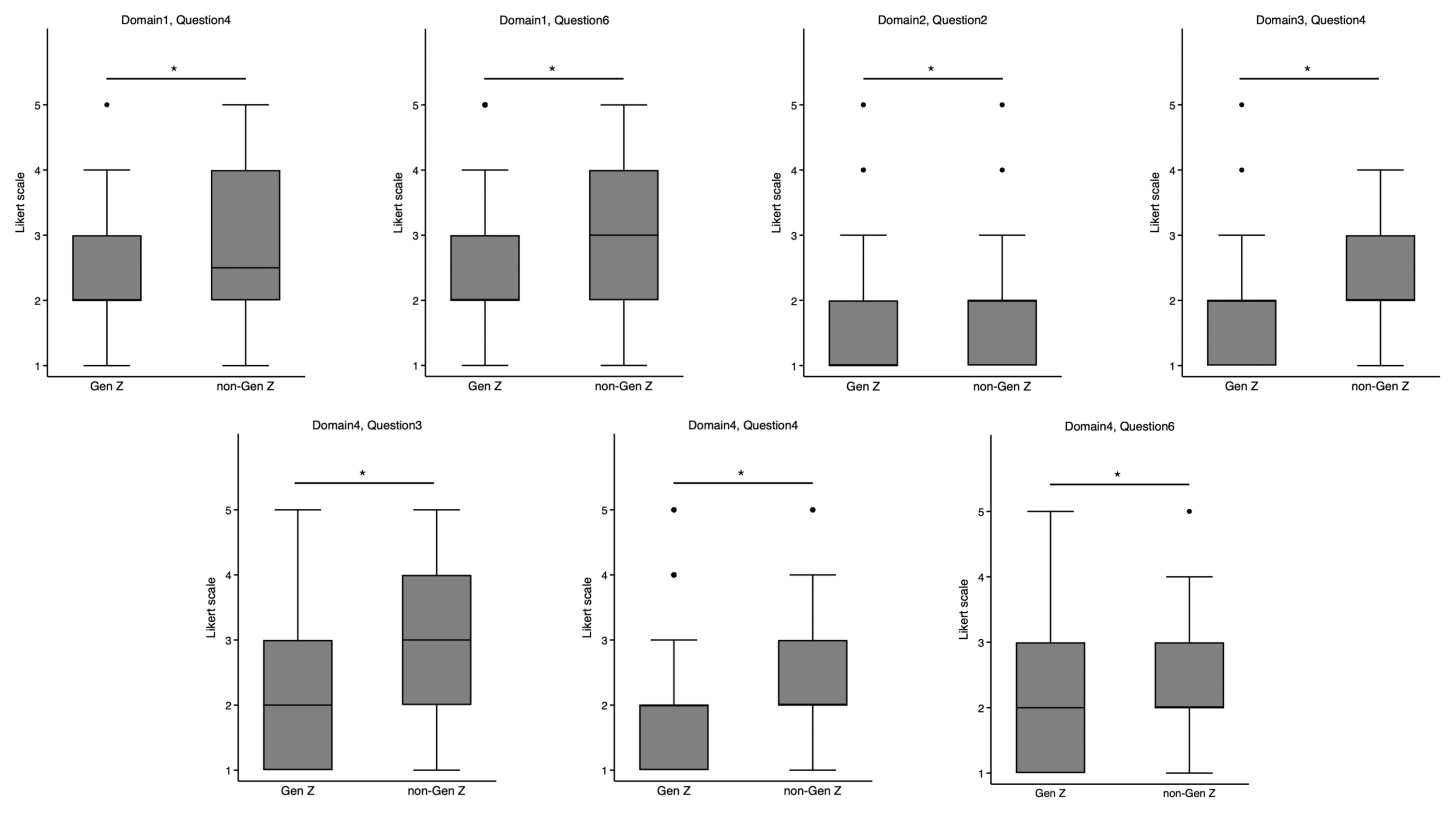
Likert scale between Generation Z (Gen Z) and non-Generation Z (non-Gen Z) physicians. Differences in median values were assessed using the Wilcoxon rank-sum test for significance. *A significant difference between Gen Z and non-Gen Z groups.

Significant differences were observed between the Gen Z and non-Gen Z groups in their responses to specific questions, as illustrated in Figure 2. Within Domain 1, differences were noted in the responses to Q4 and Q6. For Q4, the Gen Z group had a median score of 2 (interquartile range {IQR}: 2-3), whereas the non-Gen Z group had a median score of 2.5 (IQR: 2-4; p < 0.01). Similarly, in response to Q6, the Gen Z group reported a median score of 2 (IQR: 2-3) versus a median score of 3 (IQR: 2-4) in the non-Gen Z group (p = ​​0.02). In Domain 2, Q2 had different responses between the two groups. The Gen Z group recorded a median score of 1 (IQR: 1-2), whereas the non-Gen Z group reported a median score of 2 (IQR: 1-2; p < 0.01). For Domain 3, there was a difference in responses to Q4. The Gen Z group had a median score of 2 (IQR: 1-2) as opposed to a median score of 2 (IQR: 2-3) in the non-Gen Z group (p < 0.01). Finally, within Domain 4, the responses to Q3, Q4, and Q6 differed between the groups. For Q3, the Gen Z group reported a median score of 2 (IQR: 1-3) compared to a median score of 3 (IQR: 2-4) in the non-Gen Z group (p = 0.01). For Q4, the Gen Z group had a median score of 2 (IQR: 1-2), whereas the non-Gen Z group had a median score of 2 (IQR: 2-3; p = 0.01). In response to Q6, the Gen Z group had a median score of 2 (IQR: 1-3), in contrast to a median score of 2 (IQR: 2-3) in the non-Gen Z group (p = 0.04).

**Figure 3:**
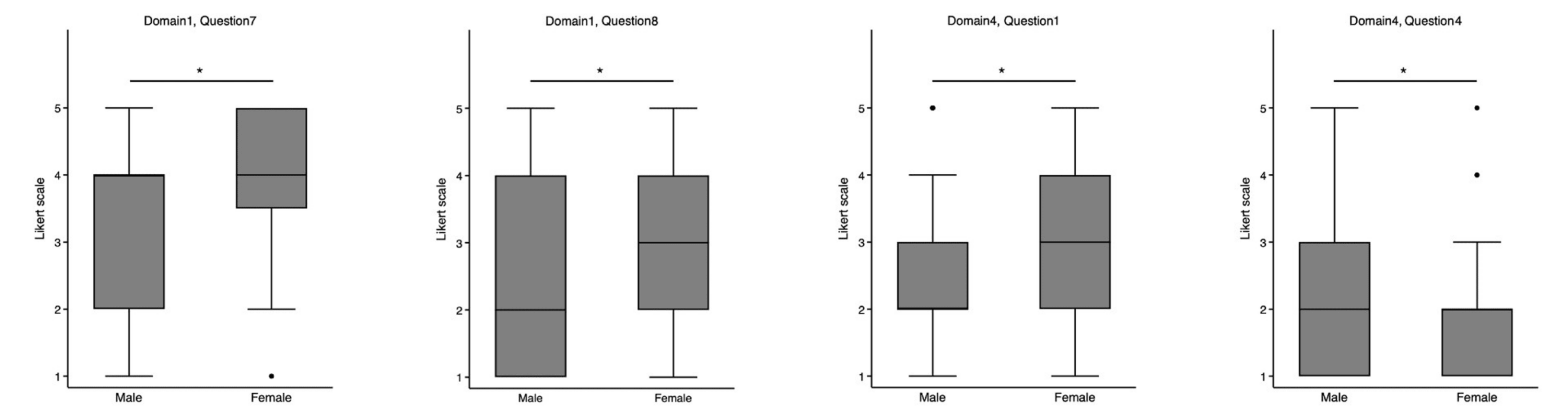
Gender-based comparison of the Likert scale within the Generation Z physician group. Differences in median values were assessed using the Wilcoxon rank-sum test for significance. *A significant difference.

Significant gender differences within the Gen Z group were observed in the responses to several questions (Figure 3). Domain 1 showed significant differences in the responses to Q7 and Q8. For Q7, the median response for male participants was 4 (IQR: 2-4), compared to 4 (IQR: 3-5) for female participants (p < 0.001). For Q8, the median response for male participants was 2 (IQR: 1-4), compared to a median of 3 (IQR: 2-4) for female participants (p = 0.02). In Domain 4, there were significant gender differences in the responses to Q1 and Q4. For Q1, the median response for male participants was 2 (IQR: 2-3), compared to 3 (IQR: 2-4) for female participants (p < 0.01). For Q4, the median response for male participants was 2 (IQR: 1-3), whereas for female participants, it was 2 (IQR: 1-2; p < 0.01).

## Discussion

Our survey of physicians in their early years of practice provides significant insights into the values and work expectations of the Gen Z cohort. These physicians show preferences for intrinsic motivation, collaborative work, instant feedback, digitalization, and communication.

In this study, Gen Z physicians emphasized meaningful work, showing intrinsic motivation. Over 70%-80% of them agreed with the importance of new challenges and acting for the benefit of others. These findings are consistent with Gen Z’s general tendency to value self-transcendence, emphasize universalism and benevolence, and be open to change, as previously reported [7]. This study also found that Gen Z physicians were significantly more likely to view work as a means to finance their personal lives. This dichotomous finding reflects Gen Z physicians’ realistic views on balancing intrinsic motivation with economic stability. Socioeconomic experiences such as 9/11 and the financial crisis of 2007-2008 have instilled realistic values for survival and success [9,10]. A previous report also suggested that Gen Z healthcare students are realistic, concerned about the future, and cautious [4]. Another professional value that Gen Z members emphasized more than non-Gen Z members was a preference for team play over individual actions. This reflects the characteristics of Gen Z, which are often described as the need for psychological security and a sense of belonging [3,4,11]. Few previous studies have focused on Gen Z physicians, with remuneration and work-life balance only reported as factors influencing their choice of specialty [12]; therefore, our study provides new insights into their professional values. Balancing certain aspects, such as intrinsic motivation, psychological safety, and financial rewards, will meet their needs. For example, factors that promote intrinsic motivation in young physicians include a self-handling environment and role models close to their age [13].

Regarding the educational aspects, Gen Z physicians preferred simulation training and instant feedback. This generation has a faster pace of life and prefers visual and kinesthetic learning. They are constantly exposed to technological stimuli and tend to seek instant gratification [4]. In addition, the “fear of missing out” (FOMO) characteristic of Gen Z, the fear of being left behind from trends or not moving fast enough in the right direction, contributes to their desire for instant feedback [9]. The average attention span of this generation is reported to be approximately six minutes, which is even shorter when using digital devices. Immersive sensory learning experiences are essential for effective education [3]. Traditional classroom lectures are not the most appropriate educational approach for Gen Z physicians. Our study also found a significant preference among Gen Z physicians for supervisors who were active communicators. This preference is consistent with previous literature indicating that Gen Z prefers approachable and passionate communicators in their educational roles [4]. Therefore, our study reaffirms the importance of a work environment that encourages active communication, particularly among Gen Z physicians.

In terms of the work environment, Gen Z physicians in this survey showed a preference for using chat rather than email for work-related communication, unlike their non-Gen Z counterparts. This preference relates to comfort through the integration of technology into many aspects of life, including work. Frequent exposure to digital stimuli and short attention spans influence their preference for chat-based communication [4]. They thrive on instant communication. Previous research has shown that 75% of Gen Z members are dissatisfied with emails as a communication medium [3]. Gen Z members may wonder why they should use emails when texting is much faster [5]. However, they may need to become more familiar with email to avoid missing important communications [3]. Some have suggested that Gen Z needs help in understanding the formalities of email communication, such as formal language, complete sentences, and the prohibition of emoticons [5]. Although supervisors of Gen Z physicians may feel that their digital communication methods are rude and uninformed, it is worth considering the merits of Gen Z’s accustomed way of communication and how to educate this generation about digital communication.

This study also found significant gender differences among Gen Z physicians in four areas: achieving results at the expense of others, wanting to diversify future careers, wanting to share personal matters at work, and preferring cooperation over competition. Men were more likely to agree with the first three items, while women were more likely to collaborate. The mention of gender differences among Gen Z physicians is a novel finding of this study. However, we are careful not to excessively overgeneralize or stereotype based on these gender findings because doing so overlooks the diversity and complexity of individuals.

This study has some limitations. First, our study involved only a limited number of convenience samples from one country, which could skew the results due to unique cultural and societal influences. In addition, considering the sample size, our study cohort of approximately 400 physicians is modest compared with approximately 30,000 physicians aged 29 years or younger in Japan, as reported by the Ministry of Health, Labour and Welfare in its 2020 statistics [14]. Furthermore, the number of participants in the non-Gen Z group was lower. There are limitations in generalizing the findings to the entire population of physicians, even in Japan. This imbalance occurred because we intentionally recruited participants based solely on post-graduate year, rather than age, to standardize workplace experiences and allow meaningful comparisons across generations. Second, the use of self-report questionnaires could have introduced bias. Self-reporting can be subjective, and the respondents can potentially provide socially acceptable answers rather than their true feelings. Finally, it is essential to consider that generations are diverse. Our study highlights differences between Gen Z and non-Gen Z physicians. However, individual factors, such as gender, socioeconomic status, education, and ethnicity, influence work values and expectations within generations.

## Conclusions

While Gen Z members valued intrinsic motivation, they were significantly more likely than members of other generations to view work as a means of earning money for their personal lives. The need for instant feedback and a significant preference for chat over email were highlighted more often among younger generations. As digital natives, Gen Z’s constantly stimulating environment has made them accustomed to instant communication, which underscores the need for integrating efficient, responsive digital communication tools into workplace training and daily practices. Additionally, the significant preference for “team” over “individual” behavior and for a communicator-type supervisor suggests that establishing psychologically safe, collaborative environments with proactive, engaging mentorship is essential for successfully guiding Gen Z physicians. Thus, medical educators and healthcare organizations should thoughtfully adapt their professional development strategies and workplace cultures to effectively engage and retain this new generation of physicians.
